# Effectiveness of traditional augmentation methods for rebar counting using UAV imagery with Faster R-CNN and YOLOv10-based transformer architectures

**DOI:** 10.1038/s41598-025-18964-1

**Published:** 2025-09-29

**Authors:** Seunghyeon Wang

**Affiliations:** Institute for Environmental Design and Engineering, University College London, London, WC1H 0NN UK

**Keywords:** Rebar inspection, Unmanned aerial vehicle, Artificial datasets, Image augmentation, Deep learning, Faster R-CNN, YOLOv10, Computational biology and bioinformatics, Engineering, Mathematics and computing

## Abstract

Accurate inspection of Reinforced Concrete (RC) structures requires precise rebar counting. Although deep-learning object detectors can extract this information from drone imagery, their effectiveness depends on large, diverse, and well-labeled datasets. Image augmentation can increase data variability, yet its impact on Unmanned Aerial Vehicles (UAVs)-based rebar counting has been underexplored. This study systematically evaluates ten augmentation methods—brightness, contrast, perspective, rotation, scale, shearing, translation, blurring, a probabilistic sampling policy, and a sum of techniques composition—using Faster R-CNN and YOLOv10 across six backbones (ResNet-101, ResNet-152, MobileNetV3; ViT, PVT, Swin Transformer). Performance is reported using AP50, AP50:95, and exact-count accuracy. Results show that augmentation efficacy is both architecture and metric-dependent. The best test-set configuration is YOLOv10–PVT with shearing, which achieves AP50 = 87.71%, AP50:95 = 68.53%, and rebar-count accuracy = 86.27%—improvements of + 5.92, + 9.07, and + 5.99 percentage points, respectively, over the PVT original baseline. A probabilistic sampling policy provides consistent, policy-level gains over original data and approaches the best single transform (especially with a magnitude ramp), whereas indiscriminate a sum of techniques application does not reliably outperform the top single augmentation.

## Introduction

Over recent decades, Reinforced Concrete (RC) columns, primarily composed of concrete reinforced with steel rebars, have gained extensive use in construction. These columns are engineered to withstand vertical loads from roofs, floor slabs, or ceilings, subsequently transferring these loads via beams to floors or foundations^1,2^. Although concrete effectively resists compressive and shear forces, its tensile strength is relatively limited. Consequently, rebars are incorporated to significantly improve concrete’s capacity to withstand tensile stresses.

The quantity of rebars embedded within RC columns critically affects both structural stability and moment resistance. Nonetheless, excessive reinforcement can escalate construction expenses unnecessarily. To ensure optimal structural performance, numerous countries have established construction codes defining the requisite number of rebars, including standards such as BS4449 in the United Kingdom^3^ and ACI 318 in the United States^4^.

Traditionally, counting rebars involves manual inspection, a method that is both labor-intensive and time-consuming. Recently, the adoption of Unmanned Aerial Vehicles (UAVs) equipped with standard RGB cameras has improved this process by allowing quicker and safer inspections through remote visual analysis. RGB imagery provides adequate detail for precise rebar counting and proves more economical than alternative sensing technologies, such as laser scanners or depth-sensing cameras^5^. Despite these advantages, manual analysis of UAV-captured images remains resource-intensive and prone to human error.

Integrating deep learning-based object detection methods presents a viable solution for automating rebar counting tasks. Object detection algorithms identify and classify specific targets within images, typically by enclosing them in bounding boxes^6^. Through appropriate training, deep learning models can predict bounding boxes corresponding to individual rebars, enabling automatic counting. As a data-driven approach, deep learning techniques efficiently manage various imaging conditions, including inconsistent lighting and differing scales. However, the effectiveness of these models is highly dependent on the availability of extensive and varied datasets, which are often challenging and resource-intensive to compile^7–10^.

To address the challenge of insufficient diversity within datasets, image augmentation provides a practical method for expanding existing data without the need for additional image collection. This approach involves applying various transformations to current images, including rotation, scaling, flipping, and adjustments to color and lighting conditions, thereby emulating a wide array of realistic scenarios^11,12^. Augmentation can significantly improve a model’s ability to generalize by introducing a more comprehensive variety of image features.

However, improper augmentation that generates unrealistic or overly artificial variations may negatively impact model performance, increasing computational requirements and reducing the importance of essential features^13–15^. Consequently, selecting augmentation techniques that effectively mimic real-world conditions is crucial to achieving optimal performance.

This study investigates several image-augmentation methods for drone-captured imagery, using deep-learning object-detection models—Faster Region-Based Convolutional Neural Network (Faster R-CNN) and You Only Look Once, version 10 (YOLOv10)—to improve rebar-counting accuracy. Both original and augmented image datasets are created to evaluate the effectiveness of these techniques. The research concludes by comparing model performances to highlight the impact of employing the recommended augmentation strategies.

## Related work

### Rebar counting-related studies

Deep learning is increasingly used for rebar counting. Prior studies have deployed diverse architectures to count rebars on construction sites^16–19^, in warehouses^20^, and in factory settings^21,22^, reporting good generalization across environments. To date, Wang et al.^5^ appear to be the only work that applies Faster R-CNN specifically to counting rebars in RC columns.

More broadly in construction-vision tasks, modern object detectors have shown strong performance, underscoring their relevance to structural component analysis. For crack analysis, Ye et al.^23^ used YOLOv7 for crack detection, and Ye et al.^24^ extended it to instance segmentation. Robust component recognition has also been demonstrated: Li et al.^25^ reported strong results with YOLOv8 for detecting prefabricated laminated slab components; YOLOv9 proved effective for prefabricated composite slab detection^26^; and Yuen and Ye^27^ showed YOLOv10 outperforming earlier YOLO versions (v5–v8).

However, within the specific context of rebar counting in RC columns, the potential gains from data augmentation remain under-explored. In particular, despite the promise of Faster R-CNN and the latest YOLOv10, their improvement through augmentation strategies has not been systematically investigated. This study addresses that gap by evaluating how traditional augmentation methods affect rebar-counting accuracy.

### Studies related to image augmentation

Recently, substantial attention has been directed toward the integration of image augmentation techniques with deep learning methodologies to tackle various construction-related problems. There is a consensus that applying diverse augmentation strategies can significantly enhance the performance of deep learning models.

Meng et al.^28^ introduced an automated system to classify rural building characteristics. Although they employed augmentation to counteract data biases, specific augmentation techniques were not explicitly detailed. Their model attained high accuracy, reporting 95.86% for abandoned structures and 99.47% for building stories. Illc et al.^29^ proposed a novel method to identify indicators of gentrification through sequential street view images, employing augmentation techniques such as flipping, shearing, and shifting, resulting in a 95.6% accuracy rate. Similarly, Boiarov et al.^30^ developed a landmark recognition system leveraging augmentation techniques like random cropping, jittering, and flipping, achieving a notable accuracy of 97.5%. Wang et al.^5^ also contributed by developing a deep learning model for counting rebars in RC columns, integrating augmentation methods including adjustments in brightness, scaling, blurring, rotation, and perspective transformations. They observed an accuracy improvement to 94.74% from 89.91% when comparing augmented datasets to original images.

While these studies emphasize the efficacy of augmentation methods, they often do not thoroughly analyze the contributions of individual augmentation techniques. Literature from various fields indicates that simultaneously applying multiple augmentation strategies does not always yield superior performance compared to utilizing single techniques independently^31^.

Dedicated research efforts have assessed the effectiveness of specific augmentation methods. For instance, Wang et al.^32^ employed image augmentation techniques, including brightness adjustments, scaling, and weather simulation, for classifying window states on building facades. These individual methods yielded accuracy scores of 92.43%, 92.84%, and 92.68%, respectively, whereas a combination of all techniques achieved a slightly lower accuracy of 92.23%. Claridades et al.^33^ evaluated augmentation approaches for detecting building entrances from street-level imagery, finding that rotation alone boosted accuracy to 85.92%. Combining Gaussian blur, brightness adjustment, and horizontal flipping further enhanced accuracy to 86.27%, and incorporating all methods resulted in an accuracy of 87.42%.

Collectively, these findings reinforce the notion that image augmentation—either through individual or combined methods—consistently improves deep learning model performance beyond original datasets. These insights highlight the untapped potential of augmentation techniques for unexplored tasks. However, the precise effects of image augmentation techniques on drone-based rebar counting remain insufficiently explored and warrant further investigation.

## Proposed approach

This study examines how image augmentation affects the accuracy of deep-learning rebar detection and counting. Ten strategies are evaluated: brightness adjustment, contrast modification, perspective transformation, rotation, scaling, shearing, translation, blurring, a sum-of-techniques scheme, and a probabilistic sampling policy.

**Fig. 1:**
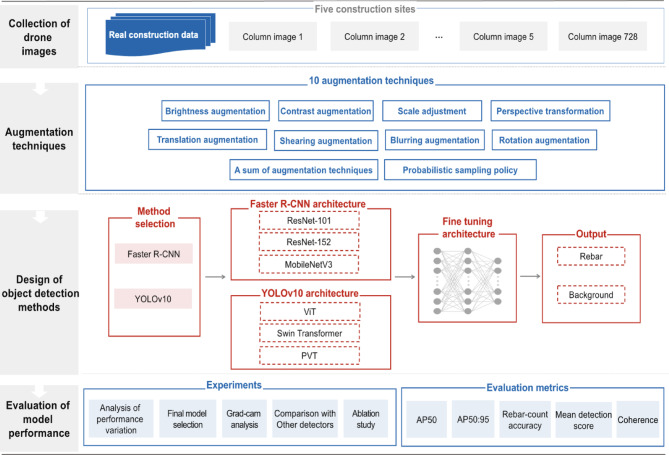
A workflow of applying the proposed augmentation methods.

The workflow depicted in Fig. 1 begins with the original dataset, upon which each augmentation technique is independently applied. Additionally, a comprehensive dataset combining all augmentation techniques is generated to further expand the diversity of the training data. Subsequently, these augmented datasets, along with the original dataset, are utilized to train deep learning-based object detection models. The performance of models trained with augmented data is then compared against those trained exclusively on the original dataset without augmentation. The sections that follow provide detailed explanations of each augmentation technique, outlining their objectives, expected benefits, and the key mathematical concepts underlying their implementation.

### Brightness augmentation

The brightness of images can vary considerably due to environmental conditions, especially when drone-captured images of rebars are acquired outdoors. For example, images obtained on sunny days generally exhibit higher brightness compared to those captured under cloudy or overcast weather conditions. To create a resilient dataset capable of encompassing such variations in illumination, brightness augmentation techniques are beneficial^34^.

Brightness augmentation artificially adjusts pixel intensity values in the original images, making them appear brighter or darker. This brightness adjustment can be mathematically represented by Eq. (1).

1 $$I^{\prime}\left( x \right) = { }I\left( x \right) + { }J\left( x \right);{ }$$

**Fig. 2:**
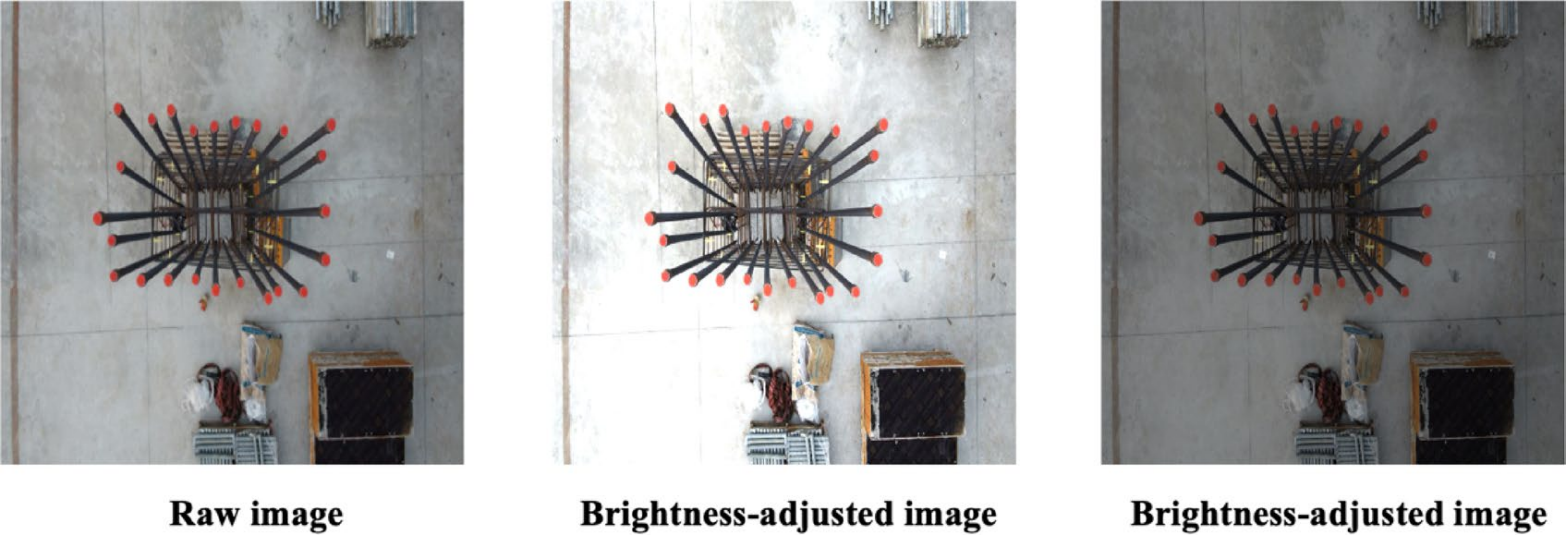
Sample images showing the effect of brightness augmentation.

In this equation, $I^{\prime}\left( x \right)$ indicates the brightness-adjusted image, $I\left( x \right)$ refers to the original image, and $J\left( x \right)$*)* represents the matrix associated with the brightness modification factor. This matrix shares the same dimensions as the original image $I\left( x \right)$. The brightness adjustment process involves adding or subtracting the $J\left( x \right)$ matrix from the pixel intensity values in $I\left( x \right),$ creating varied images that mimic different illumination conditions. Figure 2 visually illustrates this approach, showcasing examples of original and brightness-adjusted images. Specifically, the image on the left shows an increase in brightness with an adjustment value of + 30, while the image on the right demonstrates decreased brightness with an adjustment value of − 30.

### Contrast augmentation

Contrast pertains to the difference in luminance that allows objects within an image to be distinctly visible^35^. Increasing the contrast makes it easier to distinguish rebar edges from their background. From a mathematical standpoint, contrast augmentation modifies pixel intensities in proportion to the mean intensity of the entire image. The mathematical relationship for contrast adjustment is given by Eq. (2).

2 $$I_{contrast}{\prime} \left( x \right) = \alpha \cdot \left( {I\left( x \right) - mean\left( I \right)} \right) + mean\left( I \right)$$

In this equation, $I\left( x \right)$ denotes the pixel intensity at location $x$ in the original image, $mean\left( I \right)$ represents the average intensity of all pixels within the original image, and α is the scaling factor used for adjusting contrast. Meanwhile, $I_{contrast}{\prime} \left( x \right)$ signifies the contrast-adjusted pixel intensity at position $x$. The component $\alpha \cdot \left( {I\left( x \right) - mean\left( I \right)} \right)$ modifies how far each pixel intensity deviates from the average intensity. If the scaling factor $\alpha$ exceeds 1, the contrast of the image is enhanced, leading to clearer distinctions among objects. Conversely, if $\alpha$ is below 1, the contrast decreases.

**Fig. 3:**
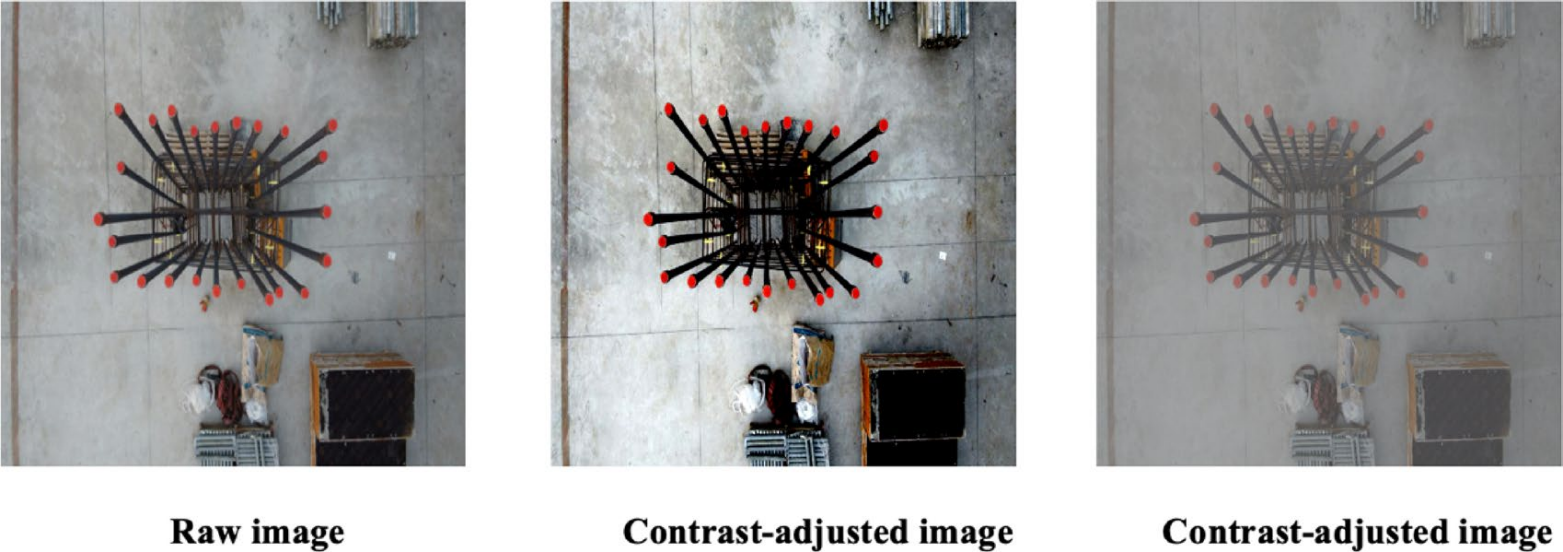
Sample images showing the effect of brightness contrast augmentation.

Examples illustrating the effect of contrast augmentation are presented in Fig. 3. The original image (left) is contrasted with two images adjusted by different scaling factors: + 1.5 for moderate enhancement (center) and + 2.6 for a more pronounced contrast enhancement (right).

### Scale adjustment

During UAV operations, altitude fluctuations cause variations in the distance between the drone’s camera and the rebar. Consequently, the rebars appear at varying scales within the captured images, either larger or smaller based on the drone’s proximity. To simulate these variations in image scale, scale augmentation is utilized. This technique involves altering the dimensions of an image by independently resizing it along both the horizontal (*x*-axis) and vertical (*y*-axis) directions, thereby producing resized variants that differ from the original image. Mathematically, the scale-augmentation transformation is given by Eq. (3).

3 $$\left( {\begin{array}{*{20}c} {x{^{\prime}}} \\ {y{^{\prime}}} \\ \end{array} } \right) = { }\left( {\begin{array}{*{20}c} {S_{x} } & 0 \\ 0 & {S_{y} } \\ \end{array} } \right) \cdot \left( {\begin{array}{*{20}c} x \\ y \\ \end{array} } \right)$$

In this equation, $x{^{\prime}}$ and $y{^{\prime}}$ correspond to the coordinates after scaling transformation in the resized image, while $S_{x}$ and $S_{y}$ represent the scale factors applied along the x-axis and y-axis, respectively, relative to the original coordinates^36^. If the scaling factors exceed 1, the image dimensions increase; conversely, factors less than 1 lead to image reduction.

**Fig. 4:**
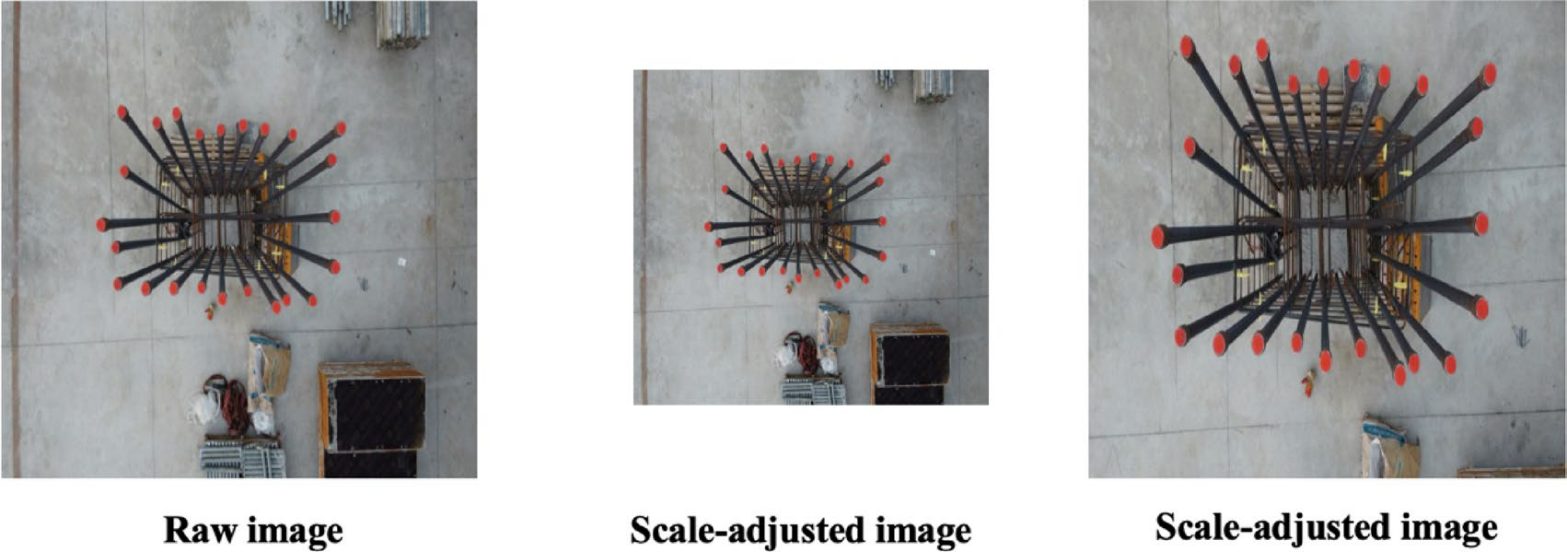
Sample images showing the effect of scale augmentation.

Figure 4 shows this process by showing examples of original and scale-augmented images. The image on the left illustrates size reduction with scaling parameters of 0.7 ($S_{x}$) and 0.8 ($S_{y}$). Conversely, the image on the right depicts enlargement through scaling parameters of 1.8 ($S_{x}$) and 2.2 ($S_{y}$).

### Perspective transformation

As UAVs capture images from varying angles during flights, the perceived orientation of rebars changes, resulting in diverse viewpoints within the collected images. Perspective augmentation utilizes a homography matrix to simulate these different perspectives, enabling trained models to more effectively recognize objects from varied angles. This transformation is mathematically defined by Eq. (4).

4 $$\left[ {\begin{array}{*{20}c} {x^{\prime}} \\ {y^{\prime}} \\ {\omega^{\prime}} \\ \end{array} } \right] = \left[ {\begin{array}{*{20}c} 1 & 0 & 0 \\ 0 & 1 & 0 \\ {a_{31} } & {a_{32} } & 1 \\ \end{array} } \right] \cdot \left[ {\begin{array}{*{20}c} x \\ y \\ 1 \\ \end{array} } \right]$$

Here, $\left( {x,{ }y} \right)$ denote the original image coordinates, $\left( {x{^{\prime}},{ }y{^{\prime}}} \right)$ represent the coordinates after transformation and is a scaling factor used for normalization. The elements and within the matrix create the perspective distortion. Normalization through ensures that the proportions and orientation of transformed images remain accurate. However, calculating the precise homography matrix directly through corner coordinates and normalization can be complex^37^.

**Fig. 5:**
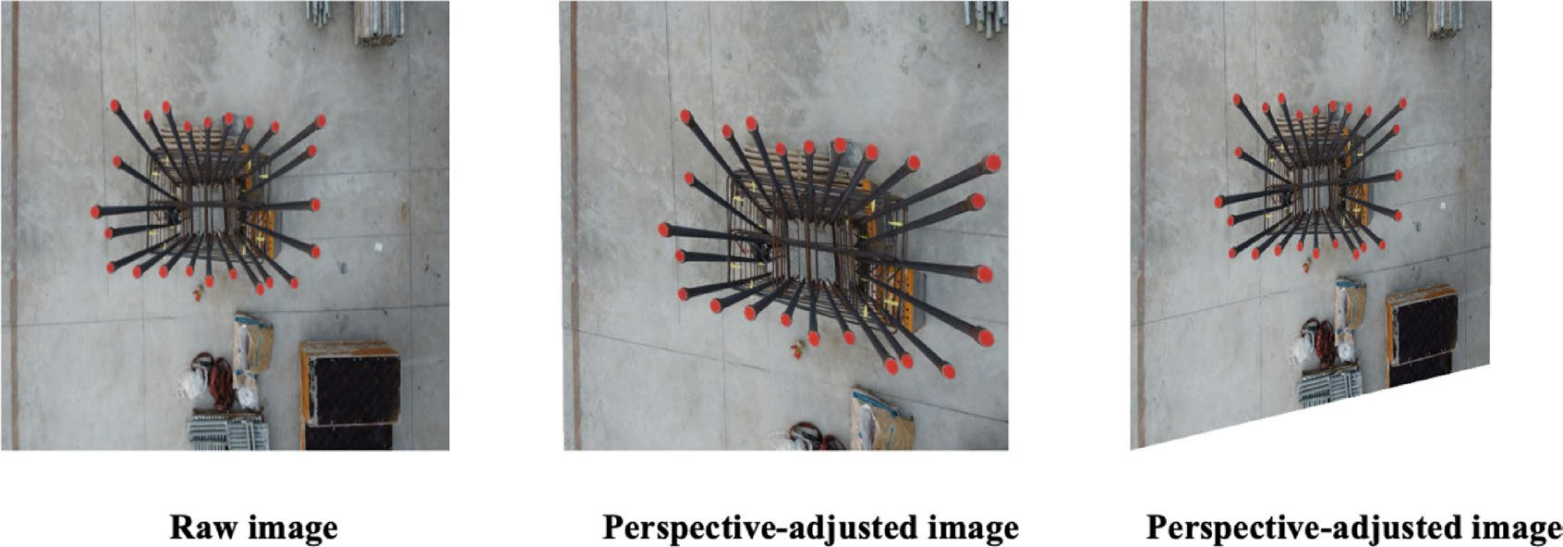
Sample images showing the effect of perspective augmentation.

To simplify this process, a practical ‘scale’ parameter specifies the magnitude of corner displacement^38^. This parameter defines the standard deviation for the random distribution from which corner shifts are drawn, eliminating the direct computation of homography matrix elements^39^. Figure 5 demonstrates the effects of perspective augmentation. The image on the left shows a perspective adjustment with a scale parameter of 0.1, while the right image applies a scale parameter of 0.17, showcasing varied perspectives.

### Rotation augmentation

During UAV operations, rotational movements cause variations in camera orientation relative to the rebar, resulting in images where rebars appear at various angles. To effectively train models capable of recognizing rebars regardless of their rotational position, rotation augmentation is applied. This technique systematically rotates original images by a designated angle. The mathematical formulation of rotation augmentation is given by Eq. (5).

5 $$I_{rotation}{\prime} \left( {x{^{\prime}},{ }y{^{\prime}}} \right) = I\left( {x\cos \left( \theta \right) - y\sin \left( \theta \right),{ }x\sin (\theta } \right) + y\cos \left( \theta \right))$$

Here, $\left( {x,{ }y} \right)$ denote coordinates in the original image, $\theta$ signifies the rotation angle, $I\left( {x,{ }y} \right)$ represents the pixel intensity at position within the original image, and $\left( {x,{ }y} \right)$ is the pixel intensity in the rotated image at the transformed coordinates $\left( {x{^{\prime}},{ }y{^{\prime}}} \right)$ ^40^.

**Fig. 6:**
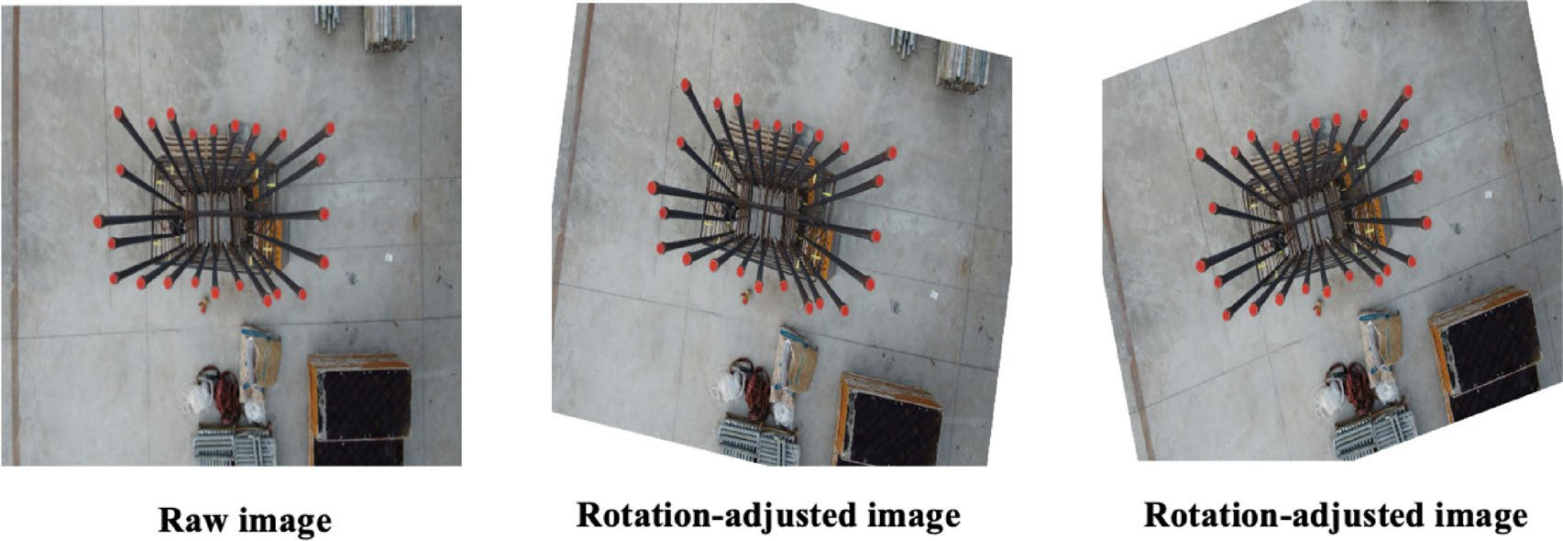
Sample images showing the effect of rotation augmentation.

Figure 6 provides illustrative examples demonstrating the effects of rotation augmentation. Specifically, the left image depicts an augmentation with a rotation angle of + 15 degrees, while the right image shows rotation at an angle of − 10 degrees. These examples showcase how rotational adjustments enhance dataset diversity, improving the model’s capability to identify rebars under various rotational conditions.

### Translation augmentation

When a UAV moves horizontally or vertically, the rebar position within captured images shifts correspondingly, causing variability in its placement within the image frame. Rebars may appear in various locations, including the image center, edges, or any intermediate position. Translation augmentation addresses this positional variation by systematically shifting the entire image content along the x and y coordinate axes, effectively enriching the training dataset^41^. The mathematical representation of translation augmentation is given by Eq. (6).

6 $$I_{translation}{\prime} \left( {x^{\prime},{ }y^{\prime}} \right) = I\left( {x - { }t_{x} ,{ }y - { }t_{y} } \right)$$

Here, $I\left( {x,{ }y} \right)$ signifies the original pixel intensity at the coordinate $\left( {x,{ }y} \right)$, $t_{x}$ and $t_{y}$ represent the horizontal and vertical translation distances, respectively, and $I_{translation}{\prime} \left( {x^{\prime},{ }y^{\prime}} \right)$ denotes the new pixel intensity at position in the translated image. By varying the values of $t_{x}$ and $t_{y}$, the direction and magnitude of image translation can be precisely controlled^42^.

**Fig. 7:**
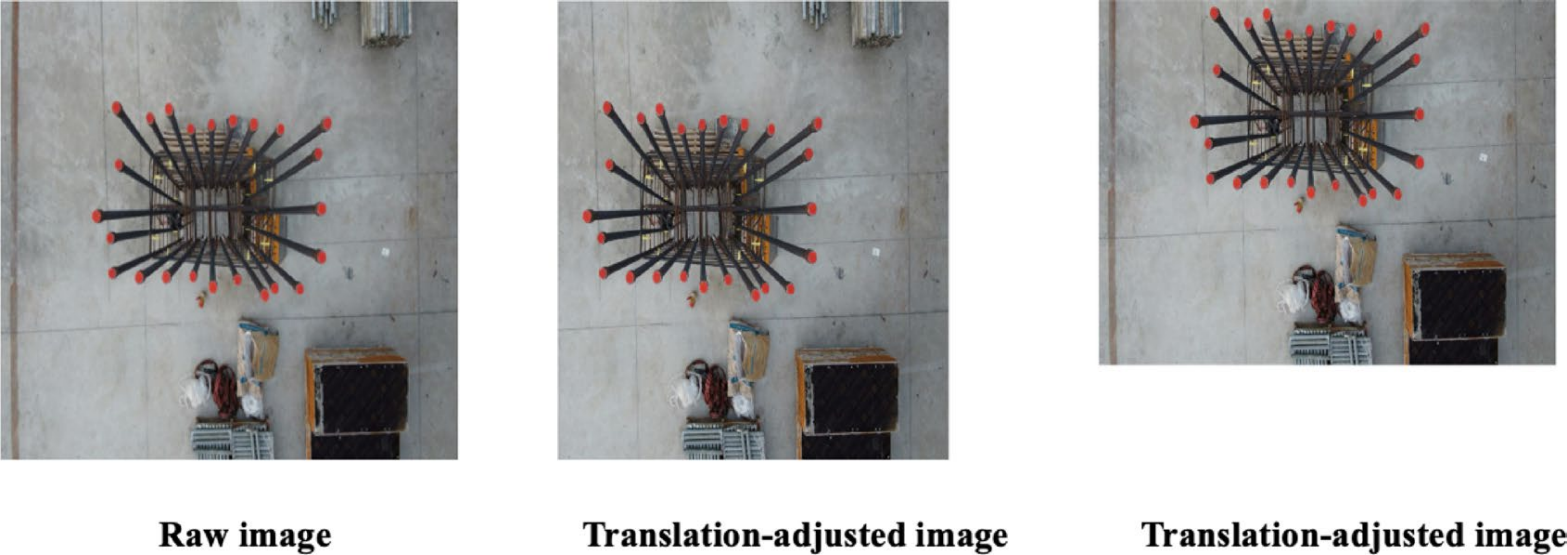
Sample images showing the effect of translation augmentation.

Examples demonstrating translation augmentation effects are presented in Fig. 7. The left image illustrates horizontal translation with parameters of $t_{x}$ = 0.7, and $t_{y}$ = 0, while the right image depicts both horizontal and vertical translation with parameters of $t_{x}$ = 1.1, and $t_{y}$ = 1.5. These illustrations emphasize how translation adjustments enhance dataset variability and support more robust model training.

### Shearing augmentation

UAV movements and variations in camera angles can result in image distortion, specifically a shearing effect, causing rebars to appear skewed or tilted. Such distortions alter the typically rectangular shape of rebars into trapezoidal forms. Shearing augmentation addresses this distortion by applying geometric transformations that systematically skew images in a specified direction^43^. This technique effectively simulates viewing the rebars from oblique angles. The mathematical expressions for shearing transformations are as follows:

For horizontal shearing, this can be defined by Eq. (7).

7 $$I_{shearing}{\prime} \left( {x^{\prime},{ }y^{\prime}} \right) = I\left( {x + sh_{x} \cdot y,{ }y} \right)$$

For vertical shearing, this can be represented by Eq. (8):

8 $$I_{shearing}{\prime} \left( {x^{\prime},{ }y^{\prime}} \right) = I\left( {x,y + sh_{y} ,{ }x} \right)$$

Here, $sh_{x}$ and $sh_{y}$ represent the horizontal and vertical shear factors, respectively. The original pixel intensity at position $\left( {x,{ }y} \right)$ is represented by $I\left( {x,{ }y} \right)$, and $I_{shearing}{\prime} \left( {x^{\prime},{ }y^{\prime}} \right)$ denotes the adjusted pixel intensity at the new coordinates $\left( {x^{\prime},{ }y^{\prime}} \right)$. By modifying the values of $sh_{x}$ and $sh_{y}$, images can be skewed horizontally (left or right) or vertically (up or down).

**Fig. 8:**
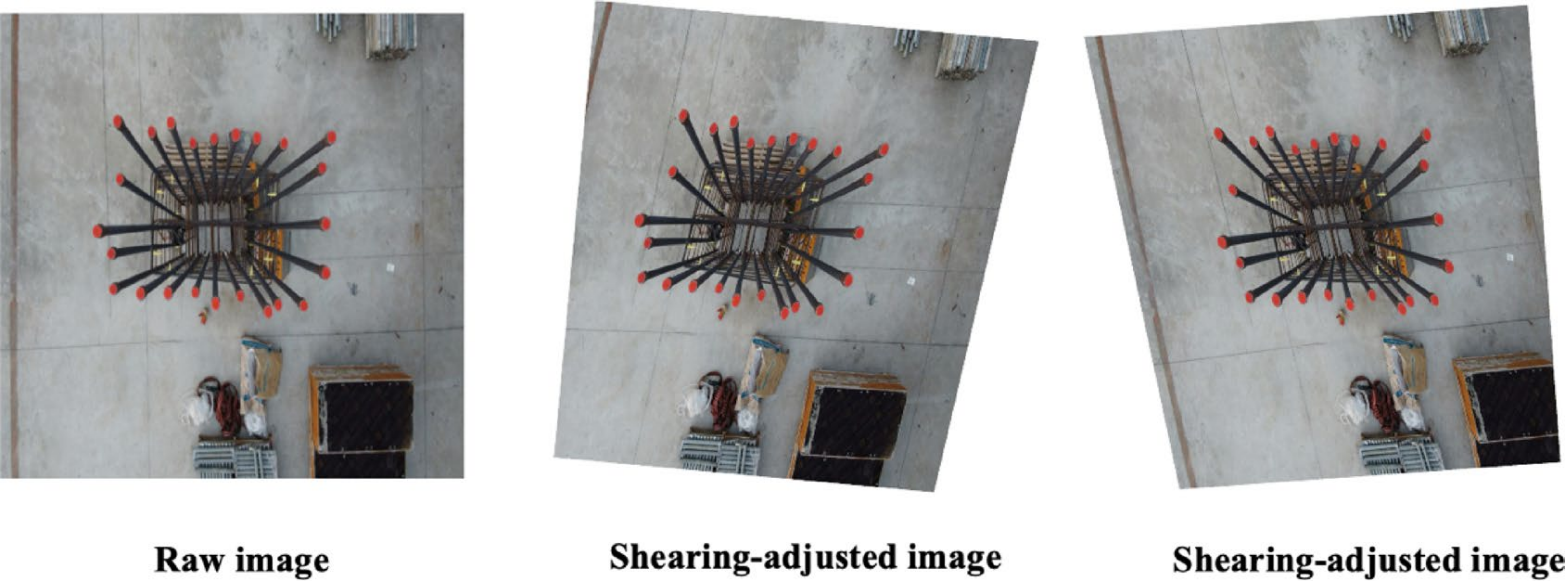
Sample images showing the effect of shearing augmentation.

Figure 8 provides visual examples demonstrating shearing augmentation effects. The left image illustrates adjustments using shear parameters $sh_{x}$ = 15and $sh_{y}$ = 20, while the right image shows a more pronounced shearing with parameters $sh_{x}$ = 20, and $sh_{y}$ = 30. These visualizations highlight how shearing augmentation effectively enriches dataset diversity, enabling models to robustly handle skewed image representations.

### Blurring augmentation

Due to windy conditions during drone flights, captured images can become blurred. Consequently, training models to accurately detect objects in blurred images becomes essential. Blurring augmentation replicates this effect artificially by applying a Gaussian filter to the original images, resulting in a smoother and less distinct appearance. Mathematically, Gaussian blurring is implemented as a convolution, as given by Eq. (9).

9 $$I_{blurred} \left( {x,{ }y} \right) = { }\mathop \sum \limits_{i = - k}^{k} { }\mathop \sum \limits_{j = - k}^{j} I\left( {x + i,{ }y + j} \right) \cdot G\left( {i,j} \right)$$

where $I\left( {x,{ }y} \right)$ represents the pixel intensity at position $\left( {x,{ }y} \right)$ in the original image, $I_{blurred} \left( {x,{ }y} \right)$ refers to the pixel intensity at the same position in the blurred image, and $G\left( {i,j} \right)$ is the Gaussian kernel defined in Eq. (10).

10 $$G\left( {i,j} \right) = { }\frac{1}{{2\pi \sigma^{2} }}{\text{exp}}\left( {\frac{{i^{2} + { }j^{2} }}{{2\sigma^{2} }}} \right)$$

**Fig. 9:**
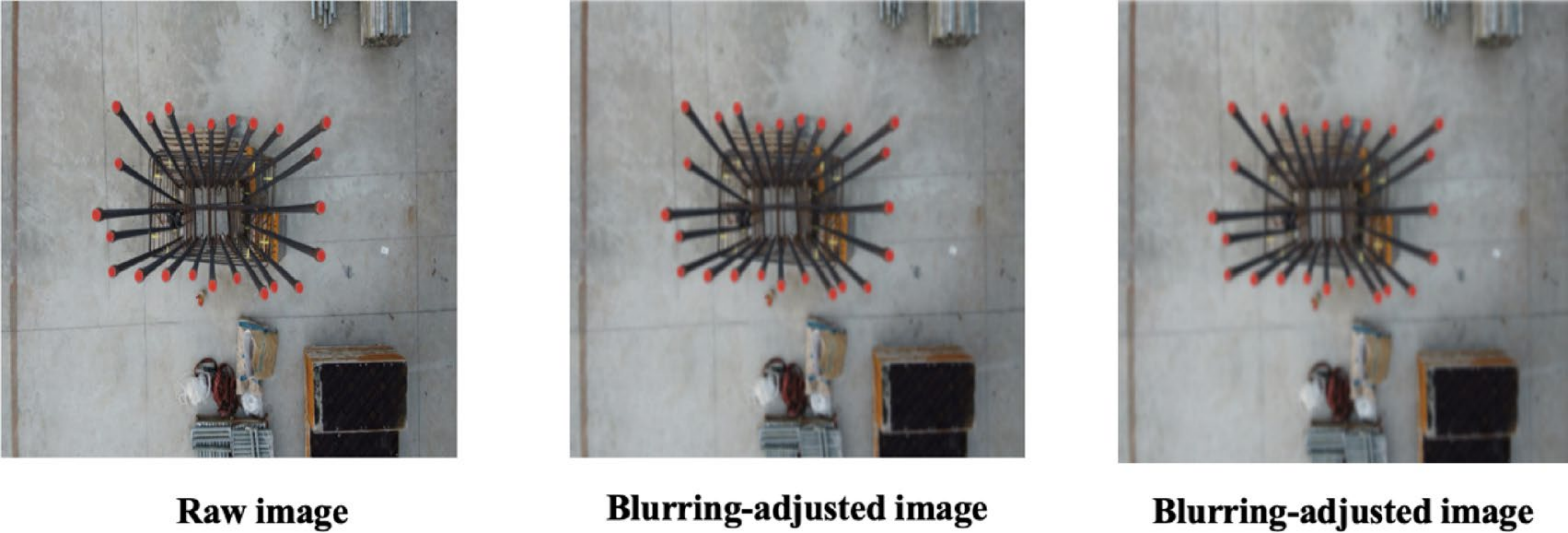
Sample images showing the effect of blurring augmentation.

The parameter $\sigma$ can be adjusted to control the degree of blurring, with higher values resulting in more pronounced blur. Figure 9 presents examples of a raw image alongside images adjusted for blurring. The left image is adjusted with a Gaussian filter parameter $\sigma$ of 0.5, while the right image is adjusted with a $\sigma$ of 1.5.

### A sum of techniques

In the “sum of techniques” scheme, one augmented copy per operator—brightness, contrast, scale, perspective, rotation, translation, shearing, and blurring—is generated from each original image. Operators are applied independently to the original (i.e., no compounding of multiple transforms on the same copy), and the training set is the union of all per-operator copies and the originals. This simple, deterministic approach, however, creates substantial redundancy and forces rare and common conditions to appear with equal frequency.

#### Probabilistic sampling policy

Probabilistic sampling uses the same augmentation operators as the “sum-of-techniques” scheme but applies them online during training. Sampling is performed strictly at the image level: for each image in each epoch, at most one geometric operator and at most one photometric operator are drawn and applied to the whole image, with magnitudes sampled from predefined ranges. Bounding boxes are not independently augmented; they are projected to the new coordinates after any geometric transform, and boxes with insufficient visibility or very small area are removed. Compared with using a single, fixed augmentation, this policy yields greater effective diversity per epoch, matches real-world prevalence by controlling transform frequencies, reduces redundancy, and avoids destructive combinations^44^—benefits expected to improve generalization, especially for thin, small objects such as rebars.

### Evaluation metrics

#### Average precision

Average Precision (AP) is extensively used as a reliable metric to assess accuracy in object detection tasks. AP quantifies precision averaged across all recall levels, ranging from 0 to 1, at different Intersection Over Union (IOU) thresholds^45^. IOU itself quantifies how well the predicted bounding boxes overlap with ground-truth boxes, defined as the ratio between the area of intersection and the area of union. Mathematically, AP is computed as in Eq. (11).

11 $${\text{AP }} = { }\mathop \sum \limits_{i = 1}^{n} p\left( i \right){\Delta }r\left( i \right)$$

Here, *n* refers to the total count of detected bounding boxes, *i* denotes the rank order of each detection, *p(i)* represents precision calculated from the initial detection to the *i*-th detection, and Δ*r(i)* is the incremental change in recall from *i* − 1 and detection *i*.

Specifically, AP50 evaluates model accuracy using an IOU threshold of 50%, effectively identifying rebars even if their bounding boxes are not precisely localized. Meanwhile, AP50:95 provides a broader assessment by averaging AP values across a series of IOU thresholds from 0.5 to 0.95 in increments of 0.05. High precision in localization, especially critical in dense scenarios, aids in accurately differentiating closely spaced rebars and minimizes the likelihood of errors due to overlapping bounding boxes. In this study, both AP50 and AP50:95 metrics are adopted to thoroughly assess model performance.

#### Rebar-count accuracy

AP is often not intuitive for engineering audiences; values such as 95.32% or 96.57% do not indicate how many rebars were miscounted. Accordingly, the primary evaluation metric is rebar-count accuracy, defined as the proportion of images for which the predicted rebar count equals the ground-truth count. For each image, the predicted count is the number of detections remaining after non-maximum suppression with a fixed confidence threshold selected using the validation set. This metric directly reflects construction-relevant outcomes—whether a column is counted correctly—and supports practical assessment of model performance for detailing, quality control of rebar installation, and site logistics.

## Experiment

### Dataset construction

#### Raw data collection

**Fig. 10:**
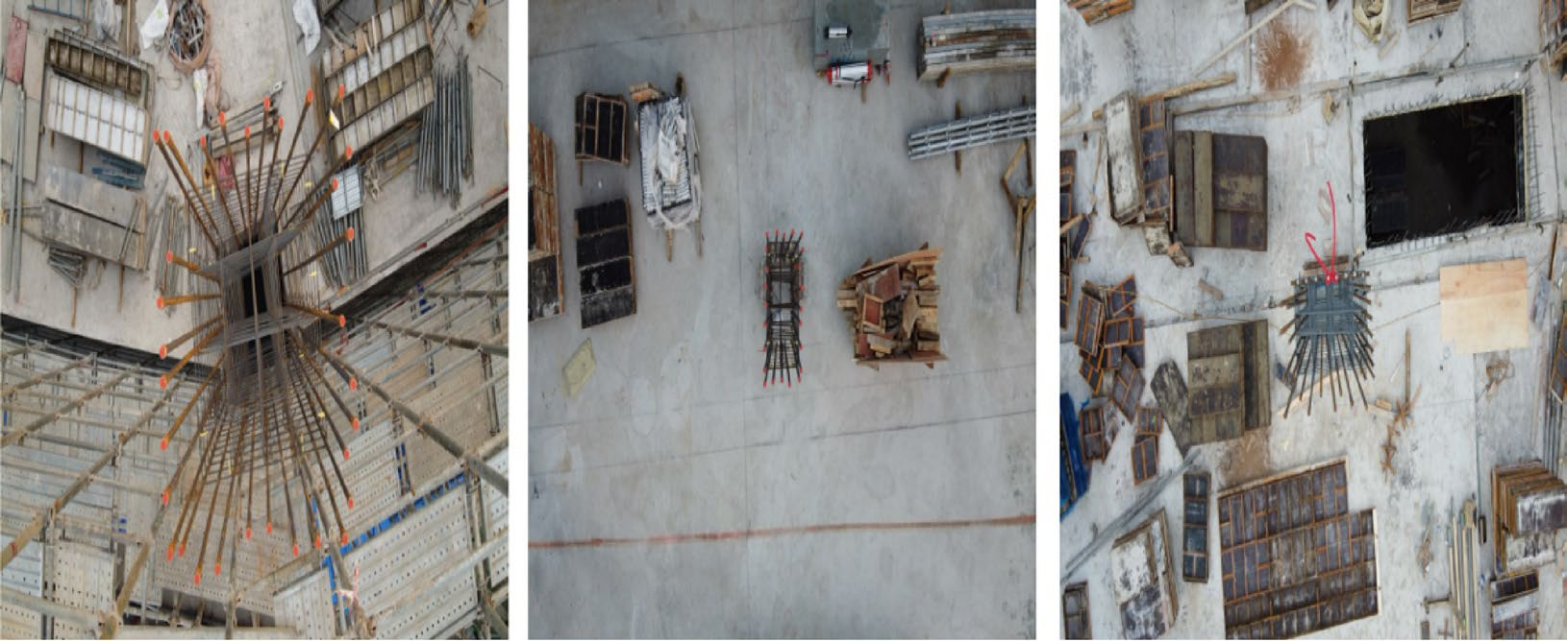
Representative samples of original dataset.

A DJI Phantom 4 Pro drone was deployed across five separate construction sites throughout South Korea during peak working hours. Inspectors operated the drone manually, capturing high-resolution, detailed imagery explicitly targeted for rebar-counting purposes. The drone was maneuvered along a strictly vertical path, consistently maintaining an altitude of 1 to 2 m directly above each rebar arrangement. At specific, preselected points, high-quality photographs were taken, positioning the columns centrally within each captured frame. To realistically evaluate the efficiency and practical applicability of the augmentation methods, images were collected under actual construction-site conditions, effectively capturing the inherent complexity present in operational settings. Examples from this original dataset are depicted in Fig. 10.

A total of 728 rebar images, each with dimensions of 1,500 × 900 pixels, were captured. The collected dataset was then split into two groups: a training subset and a test subset. Images were randomly distributed between these subsets, with 80% (582 images) assigned to the training set and 20% (146 images) designated for the test set. This random allocation ensured that both subsets proportionally represented the characteristics of the complete dataset.

#### Augmentation of training set

The effectiveness of image augmentation significantly depends on the specific parameters chosen for the augmentation techniques. These parameters were determined through an iterative trial-and-error procedure to ensure that augmented images closely resemble realistic scenarios. Table 1 provides a summary of the selected parameters and the quantities of images for each dataset.

**Table 1:** Parameter range and quantity of images in each dataset.

Configuration	Parameter range	Images	Rebars
Original (training)	–	582	14,576
Original + brightness	[− 30, 30]	1164	29,152
Original + contrast	[0.5, 2.0]	1164	29,152
Original + scale	[x: 0.8, 1.2], [y: 0.8, 1.2]	1164	29,152
Original + perspective	[0.01, 0.15]	1164	29,152
Original + rotation	[− 25°, 25°]	1164	29,152
Original + translation	[x: -0.2, 0.2], [y: − 0.2, 0.2]	1164	29,152
Original + shearing	[− 15°, 15°]	1164	29,152
Original + blurring	[0.5, 1.5]	1164	29,152
Original + Ppobabilistic sampling policy	Ranges same as individual rows	5238	131,184
Original + a sum of techniques	Ranges same as individual rows	5238	131,184
Test	–	146	3700

For brightness augmentation, pixel intensities were modified randomly within a range of − 30 to + 30 relative to the original images. Contrast augmentation involved randomly varying the alpha factor (α) within a range of 0.5–2.0. Scale augmentation resized the images independently along the horizontal and vertical axes, applying random scaling factors ranging from 80 to 120% of the original dimensions. Perspective augmentation was achieved by randomly altering the ‘scale’ parameter within the range of 0.01–0.15.

Rotation augmentation consisted of randomly rotating images within an angular range of − 25°– + 25°. For translation augmentation, horizontal (tx) and vertical (ty) shift parameters were randomly selected between − 0.2 and + 0.2 of the image dimensions. Shearing transformations applied random shear parameters (sh_x and sh_y) within a range of − 15°– + 15°. Gaussian blur augmentation was conducted by selecting random sigma σ values between 0.5 and 1.5, resulting in varying levels of blur intensity.

As shown in Table 1, eight single-technique augmented datasets were generated from the 582 training images—brightness, contrast, scaling, perspective, rotation, translation, shearing, and Gaussian blur—and one sum-of-techniques dataset that stacks all single-technique variants. In addition, a probabilistic sampling policy was used as an image-level, online augmentation regime during training: for each image in each epoch, at most one geometric and at most one photometric operator are sampled from the same parameter ranges as the single-technique datasets. Because this policy is applied online, the dataset cardinality per epoch remains 582 images (14,576 rebars). To equalize exposure with the stacked dataset, training under probabilistic sampling ran for 9 epochs, yielding the same cumulative number of image/rebar exposures as one epoch of the sum-of-techniques dataset.

#### Annotation

Each rebar within the images was labeled by drawing rectangular bounding boxes around its distinct area, forming the ground truth annotations. During this labeling process, each rebar region was specifically identified with the label ‘rebar,’ while the remaining areas were categorized as ‘background.’ Table 1 presents a detailed summary of annotated rebars in numbers for each dataset utilized in this study.

### Deep learning architecture

Within the field of deep learning-based object detection, widely adopted architectures for Faster R-CNN, such as ResNet-101 and ResNet-152, have consistently demonstrated strong performance and reliability across various detection tasks^46,47^. Additionally, YOLOv10 combined with transformer-based architectures including PVT, Swin Transformer, and ViT also exhibit robustness. However, there remains significant room to enhance their performance, particularly for specialized tasks such as rebar counting. Thus, this research selected these specific object detection methods and architectures due to their balanced trade-offs between computational efficiency and detection accuracy. For more comprehensive insights into these architectures, readers may refer to the detailed literature^48^. To facilitate accurate identification of rebars, the number of neurons in the final classification layers of these architectures was specifically adjusted to differentiate clearly between two classes: rebar and background.

### Pre-trained model through transfer learning

Developing deep learning models from scratch generally demands significant computational resources, extensive datasets, and considerable training time. A practical alternative is utilizing pre-trained models—those already trained on substantial datasets and ready for adaptation to new, related tasks^49,50^. Such models efficiently transfer previously acquired features to different applications. For this study, researchers selected ImageNet as the pre-training dataset, recognized widely for supporting academic research in computer vision^51^. Featuring over 14 million images across diverse categories, including objects such as vehicles and human figures, ImageNet provided a robust foundation for pre-training the feature extraction components within the chosen architectures.

### Optimization of hyperparameters

Effectively training deep learning models to achieve high accuracy necessitates optimizing multiple hyperparameters, as their varying configurations can considerably influence model performance^32,52^. However, this study’s primary goal is not to achieve peak accuracy through exhaustive hyperparameter tuning. Instead, it aims to assess the effectiveness of various image augmentation techniques^53^.

The models developed in this research employed specific hyperparameter adjustments. Due to GPU memory constraints and hardware limitations, the batch size was fixed at 2. Another essential hyperparameter considered was the number of epochs, a critical determinant of model performance and research validity. While reducing epochs could shorten training time, it also risked insufficiently training the dataset. Therefore, after careful consideration, the number of epochs was set at 30,000, striking a suitable balance to clearly demonstrate the impact of the proposed augmentation methods.

## Results and discussion

In evaluating the impact of augmentation techniques, evaluation metrics were presented for two different object detection models across distinct architectures, followed by an analysis of each result through discussion.

### Training and validation results

#### Faster R-CNN

**Fig. 11:**
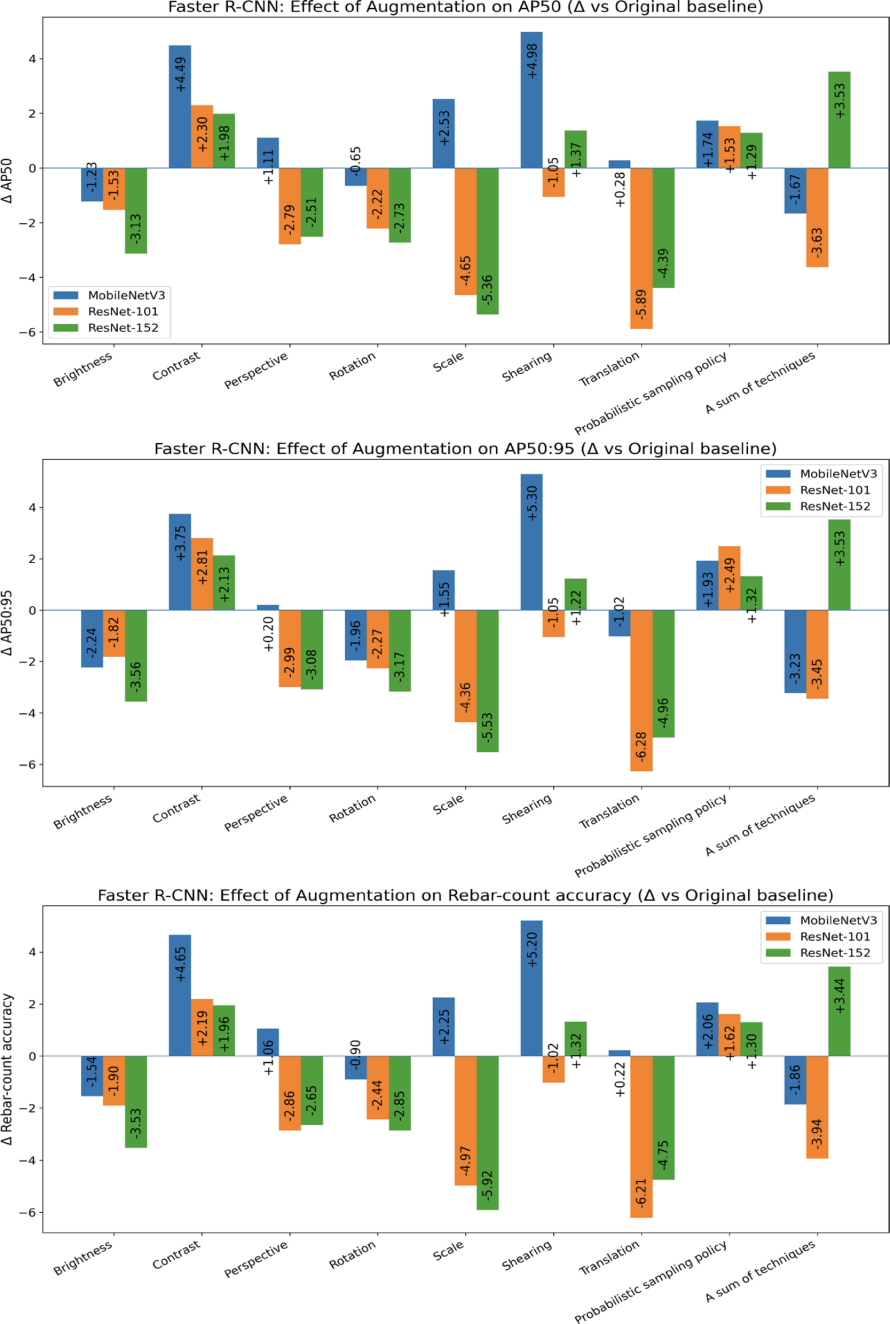
Data augmentation effects on rebar detection relative to the original baseline in Faster R-CNN.

Table 2 reports Faster R-CNN results with three backbones (ResNet-101, ResNet-152, MobileNetV3) under nine augmentation strategies, and Fig. 11 shows the change relative to the original baseline. For ResNet-101, Contrast attains the best absolute scores (AP50 = 85.87, AP50:95 = 64.45, rebar-count accuracy = 84.31), corresponding to + 2.30 / + 2.81 / + 2.19 over Original; the probabilistic sampling policy also improves all metrics (85.10/64.13/83.74; + 1.53 / + 2.49 / + 1.62).

**Table 2:** Comparison results of Faster R-CNN.

Techniques	Faster R-CNN								
	ResNet-101			ResNet-152			MobileNetV3		
	AP50	AP50:95	Rebar-count accuracy	AP50	AP50:95	Rebar-count accuracy	AP50	AP50:95	Rebar-count accuracy
Original	83.57	61.64	82.12	79.94	59.05	78.41	80.48	60.17	78.96
Brightness	82.04	59.82	80.22	76.81	55.49	74.88	79.25	57.93	77.42
Contrast	85.87	64.45	84.31	81.92	61.18	80.37	84.97	63.92	83.61
Perspective	80.78	58.65	79.26	77.43	55.97	75.76	81.59	60.37	80.02
Rotation	81.35	59.37	79.68	77.21	55.88	75.56	79.83	58.21	78.06
Scale	78.92	57.28	77.15	74.58	53.52	72.49	83.01	61.72	81.21
Shearing	82.52	60.59	81.1	81.31	60.27	79.73	85.46	65.47	84.16
Translation	77.68	55.36	75.91	75.55	54.09	73.66	80.76	59.15	79.18
Probabilistic sampling policy	85.1	64.13	83.74	81.23	60.37	79.71	82.22	62.1	81.02
A sum of techniques	79.94	58.19	78.18	83.47	62.58	81.85	78.81	56.94	77.1
Average	81.78	59.95	80.17	78.95	57.84	77.24	81.64	60.60	80.07

For ResNet-152, A sum of techniques yields the strongest absolute performance (83.47/62.58/81.85), a + 3.53 / + 3.53 / + 3.44 gain that exceeds single-technique alternatives such as Contrast (81.92/61.18/80.37). For MobileNetV3, Shearing is most effective (85.46/65.47/84.16), delivering the largest relative gains across all backbones (+ 4.98 / + 5.30 / + 5.20), with Contrast next best (84.97/63.92/83.61). Averaged across backbones, Contrast is the most reliable single augmentation (mean ΔAP50 =  + 2.92; ΔAP50:95 =  + 2.90; ΔRebar-count accuracy =  + 2.93).

In contrast, geometric Translation and Scale generally depress performance (average ΔAP50 =  − 3.33 and − 2.49), a pattern visible as negative bars in Fig. 10. The probabilistic sampling policy provides robust, moderate gains for all backbones without the instability seen when applying all techniques simultaneously, which benefits ResNet-152 but degrades ResNet-101 and MobileNetV3. Overall, photometric contrast is a safe default, shearing particularly suits MobileNetV3, and indiscriminate stacking of augmentations should be avoided except in the ResNet-152 setting.

#### YOLOv10

**Fig. 12:**
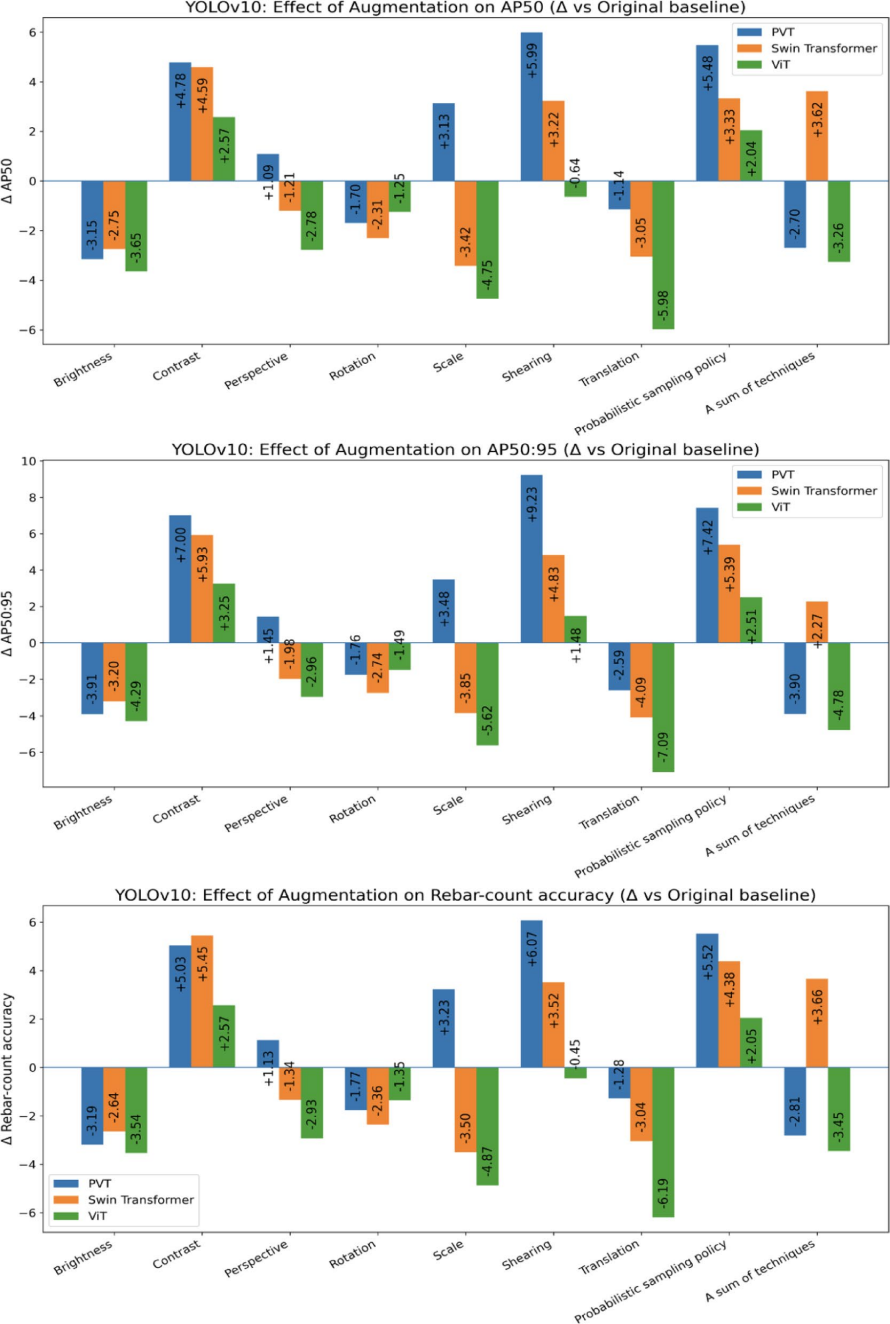
Data augmentation effects on rebar detection relative to the original baseline in YOLOv10.

Table 3 reports YOLOv10 performance with three transformer backbones (ViT, Swin, PVT) under nine augmentation strategies, and Fig. 12 shows changes relative to the original baseline. Overall, Contrast and the sampling policy are the only techniques that improve all three metrics on all three backbones: for Contrast the mean gains are ≈ + 3.98 AP50 / + 5.39 AP50:95 / + 4.35 accuracy (e.g., ViT 87.14/66.02/85.62, Δ =  + 2.57/ + 3.25/ + 2.57; Swin 84.48/64.14/83.67, Δ =  + 4.59/ + 5.93/ + 5.45; PVT 87.61/67.48/86.32, Δ =  + 4.78/ + 6.99/ + 5.03), while the sampling policy delivers similarly strong, consistent gains (mean ≈ + 3.62 / + 5.11 / + 3.98; PVT 88.31/67.90/86.81, Δ =  + 5.48/ + 7.42/ + 5.52).

**Table 3:** Results of augmentation techniques on YOLOv10 across transformer architecture.

Techniques	YOLOv10								
	ViT			Swin transformer			PVT		
	AP50	AP50:95	Rebar-count accuracy	AP50	AP50:95	Rebar-count accuracy	AP50	AP50:95	Rebar-count accuracy
Original	84.57	62.77	83.05	79.89	58.21	78.22	82.83	60.48	81.29
Brightness	80.92	58.48	79.51	77.14	55.01	75.58	79.68	56.57	78.1
Contrast	87.14	66.02	85.62	84.48	64.14	83.67	87.61	67.48	86.32
Perspective	81.79	59.81	80.12	78.68	56.23	76.88	83.92	61.93	82.42
Rotation	83.32	61.28	81.7	77.58	55.47	75.86	81.13	58.72	79.52
Scale	79.82	57.15	78.18	76.47	54.36	74.72	85.96	63.96	84.52
Shearing	83.93	64.25	82.6	83.11	63.04	81.74	88.82	69.71	87.36
Translation	78.59	55.68	76.86	76.84	54.12	75.18	81.69	57.89	80.01
Sampling policy	86.61	65.28	85.1	83.22	63.6	82.6	88.31	67.9	86.81
A sum of techniques	81.31	57.99	79.6	83.51	60.48	81.88	80.13	56.58	78.48
Average	82.80	60.87	81.23	80.09	58.47	78.63	84.01	62.12	82.48

Shearing yields the best absolute results on PVT—88.82 AP50, 69.71 AP50:95, 87.36 accuracy—and the largest deltas on that backbone (Δ =  + 5.99/ + 9.23/ + 6.07), but it slightly reduces ViT’s AP50 and accuracy. Geometric Translation and Brightness generally degrade performance (e.g., ViT Translation Δ =  − 5.98/ − 7.09/ − 6.19), and Scale hurts ViT and Swin yet helps PVT (85.96/63.96/84.52; Δ =  + 3.13/ + 3.48/ + 3.23).

Applying all techniques simultaneously is backbone-dependent—beneficial for Swin (83.51/60.48/81.88; Δ =  + 3.62/ + 2.27/ + 3.66) but detrimental for ViT and PVT. Averaged over techniques, PVT attains the highest means (AP50 84.01, AP50:95 62.12, accuracy 82.48), followed by ViT and then Swin. In sum, Contrast is a safe, high-return default; the sampling policy offers robust, cross-backbone improvements; Shearing is exceptionally effective on PVT; and indiscriminate stacking of augmentations should be avoided except for Swin.

### Model evaluation in test images

For a more rigorous evaluation, we used a held-out test set of 146 images collected from previously unseen construction sites; none of these images were included in the training or validation datasets. Table 4 presents validation–test comparisons for the best augmentation per architecture in Faster R-CNN and YOLOv10. For Faster R-CNN, ResNet-101 + Contrast shows a small decline from validation to test (AP50: 85.87 → 85.43; AP50:95: 64.45 → 63.39; rebar-count acc.: 84.31 → 83.88). ResNet-152 + A-sum-of-techniques drops slightly (83.47 → 82.91; 62.58 → 62.31; 81.85 → 81.30), and MobileNetV3 + Shearing changes minimally (85.46 → 85.15; 65.47 → 64.87; 84.16 → 83.85).

**Table 4:** Validation–test comparison results (best augmentation per architecture).

Method	Architecture	Augmentation	Validation			Test			ΔAP50	ΔAP50:95	Δ Rebar count accuracy
			AP50	AP50:95	Rebar count accuracy	AP50	AP50:95	Rebar count accuracy			
Faster R-CNN	ResNet-101	Contrast	85.87	64.45	84.31	85.43	63.39	83.88	− 0.44	− 1.06	− 0.43
	ResNet-152	A sum of techniques	83.47	62.58	81.85	82.91	62.31	81.3	− 0.56	− 0.27	− 0.55
	MobileNetV3	Shearing	85.46	65.47	84.16	85.15	64.87	83.85	− 0.31	− 0.60	− 0.31
YOLOv10	ViT	Contrast	87.14	66.02	85.62	87.36	66.38	85.84	0.22	0.36	0.22
	PVT	Shearing	88.82	69.71	87.36	87.71	68.53	86.27	− 1.11	− 1.18	− 1.09
	Swin Transformer	Contrast	84.48	64.14	83.67	84.74	64.59	83.94	0.26	0.45	0.27

Within YOLOv10, ViT with Contrast improves marginally (87.14 → 87.36; 66.02 → 66.38; 85.62 → 85.84), PVT with Shearing decreases modestly (88.82 → 87.71; 69.71 → 68.53; 87.36 → 86.27), and Swin Transformer with Contrast also improves slightly (84.48 → 84.74; 64.14 → 64.59; 83.67 → 83.94). Overall, the gaps between validation and test are small across metrics, indicating good generalization; Contrast is a reliable default across architectures, Shearing is especially effective for MobileNetV3 and PVT, and stacking all techniques is beneficial primarily for ResNet-152.

### Grad-CAM analysis results

Table 5 reports Grad-CAM–based mean detection score and coherence for each architecture under the original baseline and the backbone-specific best augmentation. Adopting the best augmentation improves both metrics for every model, with larger gains for YOLOv10 backbones than for Faster R-CNN (e.g., PVT rises by ≈ + 0.06 in mean and + 0.061 in coherence, whereas ResNet-101 and ViT rise by ≈ + 0.02–0.03).

**Table 5:** Grad CAM analysis results.

Object detection method	Architecture	Original		Best augmentation		
		Mean detection score	Coherence	Method	Mean detection score	Coherence
Faster R-CNN	ResNet101	0.836	0.821	Contrast	0.859	0.843
	ResNet152	0.799	0.784	A sum of techniques	0.835	0.819
	MobileNetV3	0.805	0.79	Shearing	0.855	0.842
YOLOv10	ViT	0.846	0.831	Contrast	0.871	0.856
	PVT	0.828	0.813	Shearing	0.888	0.874
	Swin Transformer	0.799	0.782	Contrast	0.845	0.837

Coherence tracks mean detection across all cases—improvements concentrate Grad-CAM saliency on rebar regions rather than amplifying background—indicating that augmentation sharpens both spatial attention and detector confidence rather than trading one for the other. The strongest single configuration is YOLOv10–PVT with Shearing (≈ 0.89 mean, 0.87 coherence), followed by YOLOv10–ViT with Contrast; within Faster R-CNN, ResNet-101 with Contrast slightly outperforms MobileNetV3 with Shearing.

On average, adopting the backbone-specific best augmentation increases both mean detection and coherence by approximately 0.04 relative to the original baseline, a non-negligible gain at the reported performance levels. Consequently, augmentation should be selected per backbone: Contrast for ResNet-101, ViT, and Swin Transformer; Shearing for MobileNetV3 and PVT; and combined transforms primarily for deeper CNNs such as ResNet-152.

### Ablation study

This ablation evaluates the probabilistic sampling policy on YOLOv10–PVT using the Original (no augmentation) and the backbone’s best single transform (Shearing) as reference baselines on the test set. The objective is to determine which design elements of the probabilistic sampling policy most affect performance and whether the policy approaches the single-technique baseline while clearly surpassing no augmentation.

#### Experimental setup

The probabilistic sampling policy operates at the image level, applying—per image and per epoch—at most one photometric and at most one geometric operator. Operator selection follows probability priors reflecting realistic prevalence, and magnitudes are drawn from bounded ranges. After any geometric transform, bounding boxes are re-projected and filtered by a visibility/area criterion that removes instances whose visible fraction falls below a preset threshold or whose area becomes too small, thereby avoiding supervision on near-invisible objects. The ablation varies six design choices relative to this policy: (i) replacing probability priors with uniform probabilities; (ii) restricting sampling to photometric-only or geometric-only versus using both operator families; (iii) switching from image-level to instance-level sampling, in which different objects within the same image receive different transforms; (iv) disabling the visibility/area filter; (v) forcing two operators per image instead of the “at most one per family” budget; and (vi) replacing fixed magnitudes with a ramped (curriculum) schedule over training.

#### Ablation results

Table 6 reports test-set ablations for YOLOv10–PVT. The single best transform is Shearing (AP50 = 87.71, AP50:95 = 68.53, Acc = 86.27), yielding relative gains over Original of + 7.2% AP50, + 15.3% AP50:95, and + 7.5% accuracy. The probabilistic sampling policy (image-level, one-per-family, priors, visibility/area filter) is close to Shearing (− 0.50 AP50, − 1.78 AP50:95, − 0.54 Acc) yet still improves markedly over Original (+ 6.6%, + 12.3%, + 6.8%, respectively). Adding a magnitude ramp gives the strongest policy variant, narrowing the gap to Shearing (− 0.28/ − 1.50/ − 0.31) while lifting the gains vs Original to + 6.9% AP50, + 12.7% AP50:95, and + 7.1% accuracy. Across all variants, improvements are systematically larger on AP50:95 than on AP50, indicating that augmentation primarily benefits localization quality rather than only coarse detection.

**Table 6:** Probabilistic sampling policy ablation on YOLOv10–PVT.

Configuration	AP50	AP50:95	Rebar-count accuracy	Original (baseline)			Shearing (baseline)		
				ΔAP50	ΔAP50:95	Δ Rebar-count accuracy	ΔAP50	ΔAP50:95	Δ Rebar-count accuracy
Original (baseline)	81.79	59.46	80.28	–	–	–	− 5.92	− 9.07	− 5.99
Shearing (baseline)	87.71	68.53	86.27	5.92	9.07	5.99	–	–	–
Probabilistic policy	87.21	66.75	85.73	5.42	7.29	5.45	− 0.50	− 1.78	− 0.54
Probabilistic policy + magnitude ramp	87.43	67.03	85.96	5.64	7.57	5.68	− 0.28	− 1.50	− 0.31
Probabilistic policy, uniform probabilities	86.76	66.27	85.25	4.97	6.81	4.97	− 0.95	− 2.26	− 1.02
Probabilistic policy, photometric-only	86.99	66.56	85.48	5.2	7.1	5.2	− 0.72	− 1.97	− 0.79
Probabilistic policy, geometric-only	86.38	66.03	84.96	4.59	6.57	4.68	− 1.33	− 2.50	− 1.31
Probabilistic policy, no visibility/area filter	86.58	65.89	85.06	4.79	6.43	4.78	− 1.13	− 2.64	− 1.21
Probabilistic policy, instance-level sampling	86.63	65.92	85.12	4.84	6.46	4.84	− 1.08	− 2.61	− 1.15
Probabilistic policy, force two ops/image	86.69	66.14	85.24	4.9	6.68	4.96	− 1.02	− 2.39	− 1.03

Replacing priors with uniform probabilities reduces performance (− 0.46 to − 0.76 vs the ramped policy across metrics), confirming that frequency-aware sampling matters. Restricting to photometric-only retains most of the benefit (within ~ 0.4 of the base policy), whereas geometric-only is consistently worst among the non-baseline rows (up to − 1.33 AP50 and − 2.50 AP50:95 vs Shearing), reflecting the vulnerability of thin, small objects (rebars) to geometric distortion. Disabling the visibility/area filter or switching to instance-level sampling (different transforms per object) both degrade results by roughly 0.6–1.0 points vs the base policy—consistent with label-quality loss (clipped/near-invisible boxes) and broken scene coherence. A fixed two-operator budget per image performs worse than the base policy, mirroring the instability produced by stacking transformations without restraint.

On YOLOv10–PVT, Shearing is the recommended single augmentation. When an augmentation policy is required, a probabilistic sampling policy with a magnitude ramp should be employed; image-level sampling, the visibility/area filter, and operator priors should be retained. Conversely, uniform probabilities, geometric-only variants, instance-level sampling, and forced multi-operator compositions should be avoided, as they are associated with consistent degradations in AP50, AP50:95, and rebar-count accuracy.

### Current research level of rebar counting using drone images

The current capability of the drone-based rebar-counting model is reflected in the experimental results. The best-performing configuration—YOLOv10 with a PVT backbone and Shearing augmentation—achieved AP50 = 87.71%, AP50:95 = 68.53%, and rebar-count accuracy = 86.27% on the test set, reflecting the current capability of the drone-based rebar-counting model.

**Fig. 13:**
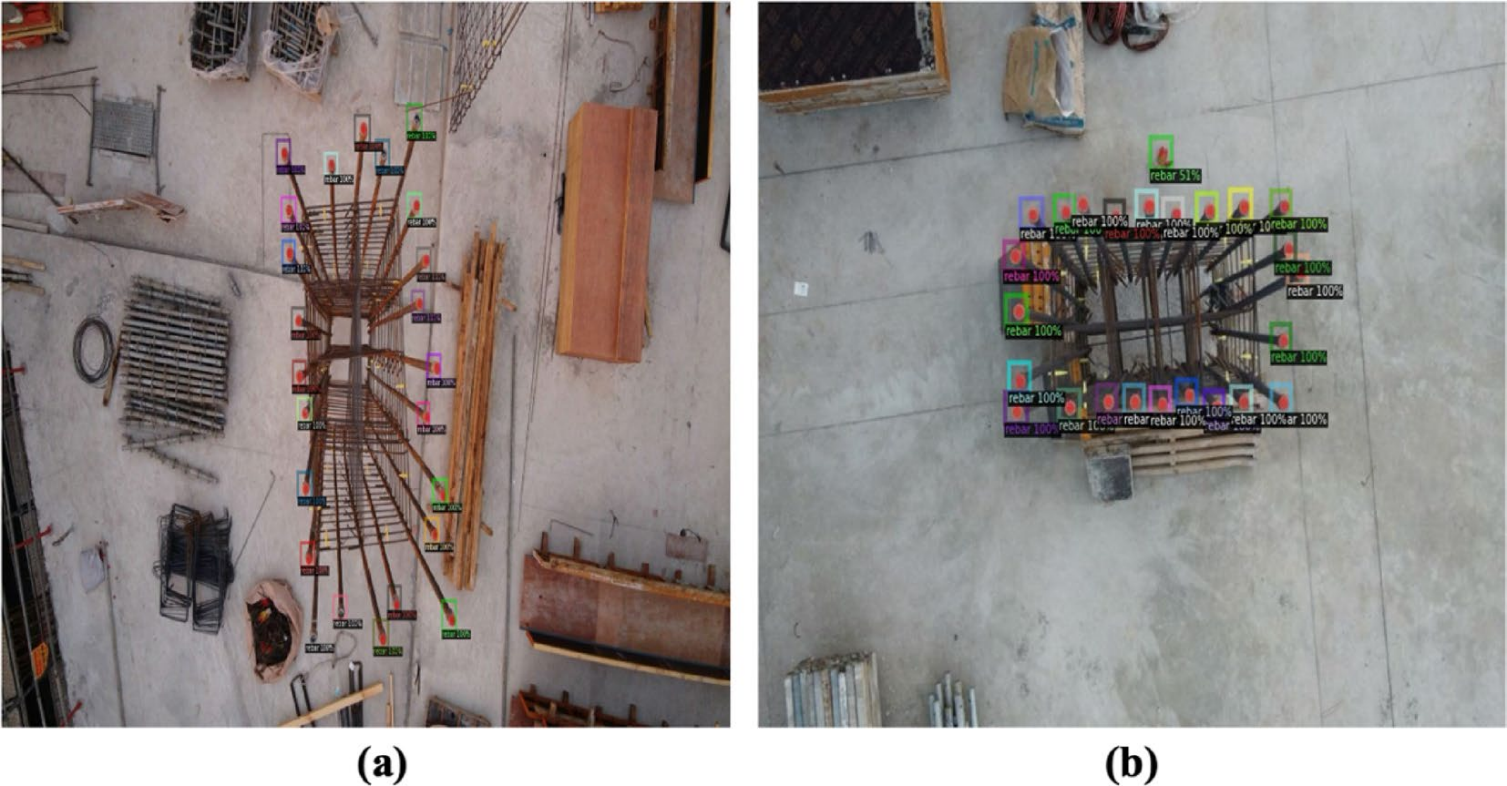
Examples of detected results from the highest-performing model.

Figure 13 illustrates examples of images with varying levels of detection accuracy. Figure 13a shows an image with high detection accuracy, where 24 rebars, mostly covered by red hoops, are visible against a complex background that includes timbers, debris, and equipment. The model successfully detected all the rebars in this image. In contrast, Fig. 13b presents a close-up view of 24 rebars covered by red hoops against a simpler background, featuring elements such as a red glove and a mold. The red glove lying on the floor near one of the rebars confused the model, leading to the glove being incorrectly identified as a rebar. This result indicates that while the model is reliable, there is still room for improvement in accuracy. Although the model achieved high accuracy, the data-driven method used in this research may not guarantee the same performance on other datasets with different data distributions. Therefore, it may be necessary to further train the model with datasets that reflect a variety of distribution patterns to enhance its robustness.

## Conclusions

This study evaluated ten image-augmentation strategies for drone-based rebar detection and counting using Faster R-CNN and YOLOv10 across six backbones. Augmentation effects were architecture and metric-dependent. Contrast emerged as the most dependable single augmentation across models, although several backbones achieved larger gains with alternative transforms (e.g., Shearing for MobileNetV3 and PVT; sum-of-techniques for ResNet-152). Averaged over backbones, moving from the Original baseline to each backbone’s best single augmentation improved performance by + 3.60 pp AP50, + 3.88 pp AP50:95, and + 3.61 pp rebar-count accuracy for Faster R-CNN, and by + 4.38 pp, + 6.14 pp, and + 4.70 pp, respectively, for YOLOv10 (ranges: Faster R-CNN + 2.30 →  + 4.98 / + 2.81 →  + 5.30 / + 2.19 →  + 5.20; YOLOv10 + 2.57 →  + 5.99 / + 3.25 →  + 9.23 / + 2.57 →  + 6.07).

The best test-set configuration was YOLOv10–PVT with Shearing, achieving AP50 = 87.71%, AP50:95 = 68.53%, and rebar-count accuracy = 86.27%, i.e., + 5.92, + 9.07, and + 5.99 percentage-point gains over the PVT Original baseline. Grad-CAM analyses showed that improvements in mean detection were accompanied by increases in coherence, indicating sharper spatial focus on rebar regions rather than a trade-off with saliency concentration. Taken together, the evidence supports backbone-specific augmentation: employ Contrast for ResNet-101, ViT, and Swin Transformer; Shearing for MobileNetV3 and PVT; and combined transforms primarily for deeper CNNs such as ResNet-152. When an augmentation policy is preferred for robustness, a probabilistic sampling policy with a magnitude ramp provides near-best performance while retaining principled control; image-level sampling, operator priors, and a visibility/area filter should be retained, whereas uniform probabilities, geometric-only variants, instance-level sampling, and forced multi-operator compositions should be avoided.

In future research, augmentation should be broadened to better reflect drone‐imaging physics and the geometry of thin structures. Promising directions include thin-structure–preserving elastic warps (e.g., small-strain or as-rigid-as-possible deformations that bound the local Jacobian to prevent rebar collapse) and physics-informed photometric models that simulate motion/defocus blur using measured PSFs, illumination and shadow geometry, haze/glare, and sensor-compression artifacts. All transforms should enforce bounding-box visibility and minimum-area constraints. In addition, generative augmentation (conditional GANs or diffusion models) can synthesize difficult cases—corrosion, occlusions, extreme obliquity—guided by masks or coarse geometry; realism can be regularized via domain discriminators or cycle consistency, and labels should be reprojected to the rendered view to avoid annotation drift.

## Data Availability

The datasets generated during and/or analyzed during the current study are available from the corresponding author on reasonable request.

## Code availability

The code developed for this study is available at the Figshare repository^54^.
